# Perspectives on supporting Veterans’ social needs during hospital to home health transitions: findings from the Transitions Nurse Program

**DOI:** 10.1186/s12913-024-10900-9

**Published:** 2024-04-25

**Authors:** Marguerite Daus, Marcie Lee, Lexus L. Ujano-De Motta, Ariel Holstein, Brianne Morgan, Karen Albright, Roman Ayele, Michaela McCarthy, Heidi Sjoberg, Christine D. Jones

**Affiliations:** 1 Denver/Seattle Center of Innovation for Veteran-Centered and Value Driven Care, VHA Eastern Colorado Healthcare System, 1700 N Wheeling St, Aurora, CO 80045 USA; 2 Pitkin County Public Health, Aspen, CO USA; 3 Department of Medicine, University of Colorado Anschutz Medical Campus, Aurora, CO USA; 4 OCHIN, Inc., Portland, OR USA

**Keywords:** Social determinants of health, Veterans, Home health care, Care transitions, Community care

## Abstract

**Background:**

Veterans who need post-acute home health care (HHC) are at risk for adverse outcomes and unmet social needs. Veterans’ social needs could be identified and met by community-based HHC clinicians due to their unique perspective from the home environment, acuity of Veterans they serve, and access to Veterans receiving community care. To understand these needs, we explored clinician, Veteran, and care partner perspectives to understand Veterans’ social needs during the transition from hospital to home with skilled HHC.

**Methods:**

Qualitative data were collected through individual interviews with Veterans Health Administration (VHA) inpatient & community HHC clinicians, Veterans, and care partners who have significant roles facilitating Veterans’ hospital to home with HHC transition. To inform implementation of a care coordination quality improvement intervention, participants were asked about VHA and HHC care coordination and Veterans’ social needs during these transitions. Interviews were recorded, transcribed, and analyzed inductively using thematic analysis and results were organized deductively according to relevant transitional care domains (Discharge Planning, Transition to Home, and HHC Delivery).

**Results:**

We conducted 35 interviews at 4 VHA Medical Centers located in Western, Midwestern, and Southern U.S. regions during March 2021 through July 2022. We organized results by the three care transition domains and related themes by VHA, HHC, or Veteran/care partner perspective. Our themes included (1) how social needs affected access to HHC, (2) the need for social needs screening during hospitalization, (3) delays in HHC for Veterans discharged from community hospitals, and (4) a need for closed-loop communication between VHA and HHC to report social needs.

**Conclusions:**

HHC is an underexplored space for Veterans social needs detection. While this research is preliminary, we recommend two steps forward from this work: (1) develop closed-loop communication and education pathways with HHC and (2) develop a partnership to integrate a social risk screener into HHC pathways.

**Supplementary Information:**

The online version contains supplementary material available at 10.1186/s12913-024-10900-9.

## Background

Veterans who have additional support needs at home with skilled home health care (HHC) after hospitalization may be at higher risk for adverse outcomes and unmet social needs compared to those in other post-acute care settings [1, 2]. For example, in facility-based post-acute care settings, housing, food, and support needs are met by the facility. Social needs are individual-level unmet material needs such as housing, food insecurity, and social support that are dependent on people’s individual preferences and priorities [3, 4]. Salient social needs identified in Veteran populations include social isolation, educational needs, interpersonal violence, housing instability, and utility concerns [5]. The specific social needs of Veterans receiving HHC remain unknown. Despite the Veterans Health Administration’s (VHA) commitment to address social determinants of health (SDOH) factors in the 2022–2028 Strategic Plan, the VHA does not yet have a universal, integrated social risk screening system across the healthcare system to detect Veterans’ social needs [6]. 

HHC is an underexplored space for social needs detection for Veterans who receive care in the community. The care transition from hospital to home is one of the most vulnerable times and patients receiving HHC have higher acuity that put them at greater risk for adverse outcomes during this period [7]. As social needs drive physical health outcomes, Veterans with social needs may have compounded risk during an already vulnerable time [3, 8]. Additionally, acute care is focused on stabilizing a patient to return to home and may not be the appropriate setting to address social needs longitudinally. Social needs-targeted care should occur in every step of the care transition, but HHC is unique in a few ways: (1) clinicians are often in the home within 48 h of discharge and the first healthcare a Veteran receives outside of the hospital to address social needs early on, (2) HHC clinicians have access to the home environment and may detect social needs not captured on a screener, (3) HHC provides multiple visits over one or more months giving clinicians time to address and follow up on social needs.

For Veterans, nearly all skilled HHC services (e.g., nursing, physical and occupational therapy) are provided by non-VHA community HHC agencies and financed through VHA-purchased care or Medicare. While coverage between VHA and Medicare HHC are similar, the VHA coverage varies by Veteran service connection and priority status. For example, the number of visits approved or coverage of a HHC social worker may differ from the Medicare benefit. Due to this structure, issues around care coordination, delays in HHC, and communication between community HHC agencies are common. These issues between VHA and community care are not unique to HHC. Many Veterans, community care clinicians, and researchers desire standardized care coordination between the VHA and the community [9, 10]. HHC agencies are frequently unfamiliar with VHA benefits and social support resources [11, 12]. They also report barriers to effective communication without standardized processes. Access to Veterans’ home environment may enable HHC clinicians to detect social needs that go unobserved in acute care settings, but it is unknown if they are detecting and reporting identified social needs to the VHA. This knowledge gap can affect Veterans’ ability to access VHA and community social needs resources.

While few researchers have explored the experiences of HHC clinicians meeting patients’ social needs, in one study, 95% of HHC nurses believed their limitations in addressing social needs resulted in their patients developing new conditions such as circulatory issues, cardiac conditions, and orthopedic issues from falls [13]. Furthermore, HHC nurses in this study thought limitations in addressing social needs increased patient acuity which contributed to higher readmission rates and suggested that addressing social needs during HHC visits could be preventative if integrated into usual HHC [13]. 

To better understand these factors during the transition from hospital to home with skilled HHC, our study examines inpatient clinician, Veteran, and care partners (partners, friends, neighbors, and others who participate in the care of the Veteran) experiences and recommendations to meet Veterans’ social needs throughout the transition from hospital to home with HHC [14]. We sought to identify the unique social needs of Veterans receiving HHC and to understand barriers and solutions to meeting social needs experienced by Veterans during these transitions. Insights from these interviews could inform future interventions to detect and address social needs during Veterans’ transitions of care.

## Methods

We conducted a secondary analysis of semi-structured interviews with inpatient clinicians from four Veteran Affairs Medical Centers (VAMCs), HHC agencies that served Veterans referred from the four VAMCs, Veterans and care partners. We conducted interviews at four sites that were planning to implement or actively implementing the Transitions Nurse Program for Home Health Care (TNP-HHC) as a quality improvement (QI) intervention, which was part of a larger VHA Quality Enhancement Research Initiative (QUERI) proposal (QUERI 20 − 013) [15]. TNP-HHC is a Veteran-centered intensive care coordination intervention to improve hospital to home with HHC transitions for Veterans. The parent study evaluated participants’ experiences with care coordination following discharge from a VAMC with HHC. The findings informed the implementation of TNP-HHC in coordination with VA and non-VA settings to increase coordinated home healthcare services to Veterans. The 4 TNP-HHC sites were geographically diverse with VAMCs located in Western, Midwestern, and Southern U.S. regions. Analysts involved in the recruitment of interviews include PhD (RA, LM, MM) and master’s level (ML, CR, MSM, GS) health service researchers with varying levels of experience in qualitative research. Interviews were conducted in March 2021 through July 2022 over Microsoft Teams meetings or phone and recorded, ranging from 12 to 72 min. All participants provided verbal informed consent. Audio recordings were saved on a protected network, transcribed, and coded in ATLAS.ti v9 [16]. 

For participants employed at VAMCs or HHC agencies, interview questions were asked about existing processes to coordinate care for Veterans requiring HHC after an inpatient discharge. For Veterans and care partners, interview questions asked about their recollection and experiences during the transition from hospital to home with HHC. All interview guides were developed by the study team and informed by the Practical, Robust Implementation and Sustainability Model (PRISM) [17]. The interviews were intended to better understand the context for a new QI intervention to improve care transitions for Veterans. Therefore, sampling was not determined by thematic saturation, but rather by the representativeness of roles of individuals involved in implementing TNP-HHC at each site. This was a secondary analysis to examine participant perspectives about Veterans’ social needs. All participants were asked about Veterans’ social needs during the time a Veteran is admitted to the hospital and discharged home with HHC (Supplement 1). Questions included asking VHA and HHC clinicians about social needs that were observed during discharge planning or HHC visits and Veterans/care partners about unmet needs they experienced after hospitalization. Follow up questions were asked about how those needs are addressed and if Veterans had resources to address unmet needs for which they desired support.

### VHA inpatient clinician, HHC clinician, and veteran/care partner recruitment

We used a convenience sample to recruit participants. The VHA project team implementing TNP-HHC at the four VAMCs provided a list of inpatient clinicians and HHC agencies involved in HHC coordination. While primary care clinicians may be involved in coordinating HHC, our study focused on post-acute care transitions to home which is ordered by inpatient clinicians. Therefore, our sampling strategy was focused on this clinician group. Veterans admitted to participating VAMCs and discharged home with HHC over a five-month period were recruited to the study by screening VHA data dashboards and validation with case managers that Veterans had received HHC. Veterans transitioned into hospice care were excluded. Emails were sent to invite potential VHA and HHC participants to interview. Interested individuals replied to the project team and were scheduled for virtual interviews. Snowball sampling was used to get additional names of possible participants involved in TNP-HHC implementation. If contact outreaches were exhausted, the project team returned to VHA case managers and discharge planners for more contacts.

### Approach & care transition domains framework

Three analysts (AH, MD, and ML) with 1–5 years of qualitative experience coded and analyzed the data to focus on how SDOH influenced the transition from hospital to home with HHC. The analysts used an inductive content analysis approach to establish an initial code list [18]. For consensus, we coded the first few transcripts together using an inductive approach from each subset of data (i.e., Veteran/care partner, VHA, HHC). Each analyst’s coding was combined in ATLAS.ti and the analysts reviewed the coding together and discuss discrepancies and refined the codebook [16]. After reaching consensus on the initial code list, analysts then coded an additional transcript to apply the agreed upon codes. As analysts continued to code more transcripts, additional codes were created to document ideas that were not depicted in previous data. Data sets were merged iteratively until all transcripts were coded. The analysts met consistently to discuss, define, and agree upon new codes and emergent themes arising from the data.

**Fig. 1:**
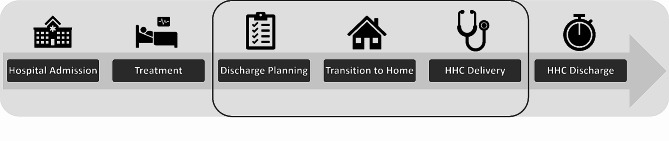
The care transition model

To organize the results of our inductive coding, we used Burke et al’s Ideal Transition in Care and Hansen et al’s care transition categories to define the care transition model from hospital to home with HHC and the 3 domains of focus for presenting the results. Figure 1 displays the 6 steps in the care transition from hospital to home with HHC and identifies the 3 domains of focus for this analysis: (1) Discharge Planning, (2) Transition to Home, (3) HHC Delivery. Using this model, we reviewed our codes and organized our results to identify how social needs are considered in each step of the transition from hospital to home with HHC from four different perspectives (VHA, HHC, Veterans, care partner) [19, 20]. We identified 9 themes from this work, one per perspective in each domain.

In accordance with VHA Institutional Review Board, the TNP-HHC study is a designated quality improvement intervention as part of the QUERI program and did not require full review. The COREQ checklist for qualitative studies was used to assure quality standards [21]. Since the parent study was designated quality improvement, informed consent was not required but we obtained verbal consent to record the audio after providing the participant an explanation of the program.

## Results

We conducted 35 interviews including 11 Veterans who recently transitioned from hospital to home, 2 care partners (1 dyad interview), 14 VHA inpatient clinicians, and 9 HHC clinicians across 7 HHC agencies who provide care for Veterans from the participating VAMCs. Roles of HHC participants included director, sales representative, and coordinator roles. Roles of VAMC participants included physician, community care nurse, and social work chief. Forty-seven VHA and HHC clinicians (36 passive, 11 active) and 35 Veterans (30 passive, 5 active) did not respond to participation requests or declined. Veteran characteristics are presented in Table 1.

**Table 1:** Description of veteran subjects

Veteran Characteristics (*n* = 11)	n (%)
Male	10 (91%)
Age (Mean, SD)	65 ± 10.5
Married	6 (55%)
Rural	5 (45%)

While the specific social needs of Veterans receiving HHC are unknown, our interviews surfaced several social needs that may be salient to this population. Together these needs provide important context for our results. Veterans and care partners described three social needs that affected their care: (1) transportation, (2) social support, and (3) finances/surprise medical bills.

Transportation: “I’ve been getting somebody else to drive for me cause… that long a drive is tough and, you know, that back-to-back and that close together… [the VHA] they would give me a little travel pay but that’s about it” – Veteran, Site 3.

Social support: “…some Veterans if they doesn’t have someone there to help them, maybe they will forget to take their medication or the appointments because somebody is there with them, but if somebody is not there, they will probably need a little help or calling on the phone reminding them that you got these pills or you’ve got an appointment or something to take.” – Care Partner, Site 1.

Surprise medical bills: “People don’t know how to do the paperwork and you end up with a bushel basket of bills… it turned over to a collection agency before I know what was going on.” – Veteran, Site 3.

**Fig. 2:**
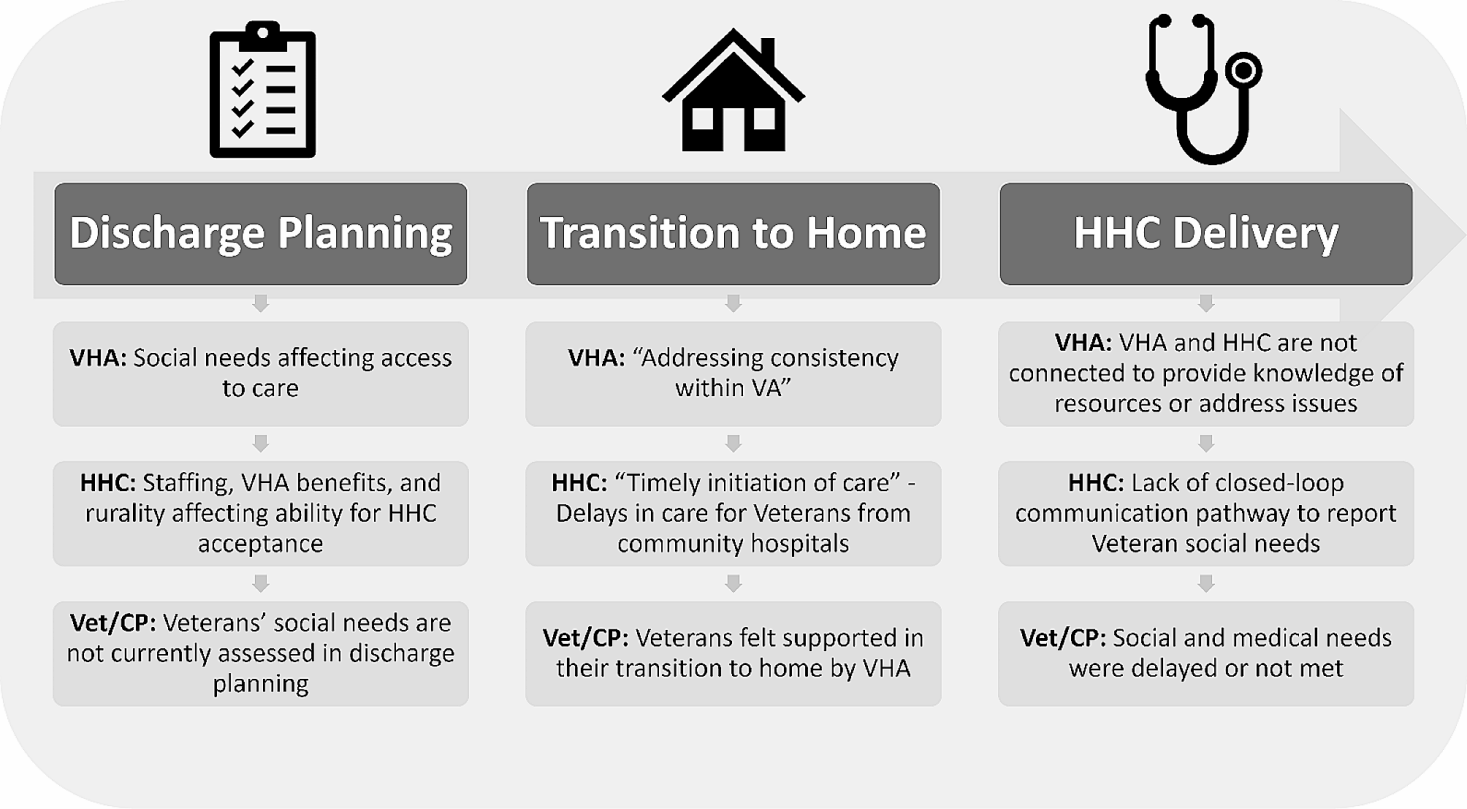
Themes by domain of the care transition model. VHA = Veterans Health Administration, HHC = Home Health Care, Vet = Veteran, CP = Care Partner

Identification of these social needs better frames our results and adds to the limited knowledge we have about Veterans’ social needs. Our results are organized by the three care transition domains with related themes presented by participant perspective due to the unique issues that arise within each domain (Fig. 2).

### Discharge planning

Many clinician participants described how higher-acuity Veterans with social needs related to rurality, transportation, and social support affect access to care for Veterans during discharge planning. Although social needs screening is not universal in the VHA, some social needs are identified by clinicians during hospital care. These social needs affect VHA community care coordinators’ ability to find HHC agencies that accept Veteran referrals and may result in nursing home placement instead of returning home:

Transportation is an issue as far as getting the Veterans home. There’s I guess caregiver fatigue or lack of a caregiver… they’re not quite ill enough where they need to go to a nursing home, but they’re not quite functional enough where they can do their own dressing changes daily and sometimes they just don’t have anyone who can help them with that at home and home health can’t come out every day or for long periods… lack of social support for some of the Veterans has been hard and has made it so that the only option then is to go to a nursing home. – VHA Physician, Site 4.

From the HHC perspective, payment and staffing affect their ability to provide HHC for Veterans. Veterans in rural areas or Veterans covered only by VHA benefits may have challenges with HHC agency placement due to staffing constraints and case mix balancing, the balancing of patient complexity and the benefits provided by different payors including VHA and Medicare. Participants from both VHA and HHC described these barriers to care:

If there is a payer source besides the VA, like Medicare/Medicaid, [Discharge Planners] will call the home health agencies and set it up, set up whatever is needed for that Veteran using that payor source. The home health agencies here prefer Medicare and private insurance over VA pay, so we try to accommodate them so that they will accept our Veterans… – VHA Community Care Nurse Manager, Site 4.

Another HHC Coordinator reported feeling “out of the loop” during discharge planning and indicated that improved communication between the VHA, HHC, and community care could be part of the solution.

Several participants across sites reported that screening for Veterans’ social needs is not integrated into VHA discharge planning (e.g., housing, food insecurity, social support) besides ensuring that Veterans have a discharge location. Veterans described a desire for inclusion in decision making and communication throughout discharge planning. One Veteran spoke about not being informed of their HHC enrollment:

I wish I would have known, like how often they need to come by, and, you know, I just wish I would have known that ‘cause nobody told me that they enrolled me in home health care. They just kind of showed up, so I had no idea. I was wondering like why they were there. I knew physical therapy needed to come by, but I had no idea that I was enrolled in home health care… – Veteran, Site 3.

### Transition to home

Both VHA and HHC participants noted inconsistent information about HHC processes for Veterans with VHA benefits during the hospital to home with HHC transition. They described situations where VHA contacts would have varying levels of knowledge about HHC or how VHA organization is different from community hospitals. One potential solution discussed by HHC partners was education for clinicians within VHA and community care (e.g., HHC, community hospitals, skilled nursing facilities) on VHA benefits and processes to better serve Veterans and navigate the VHA ecosystem:

I feel like sometimes my office will call one VA doctor who completely understands the process and says yep, we’ll do this, this and this, and then you have another one that says I don’t know what you’re talking about… So, maybe addressing that consistency within the VA side, but also educating civilian hospitals about that process. – HHC Coordinator, Site 1.

HHC agencies across sites described delays in care for Veterans discharged home from community hospitals. Many remarked that they lacked a communication pathway to easily access VHA clinicians for HHC orders, leading to delayed authorization and HHC delivery. One HHC coordinator voiced these concerns:

If a patient is at an outside hospital, and they have a need for home care, it shouldn’t take five days for that patient to have home care started… if it’s hung up waiting in queue at a doctor’s office for a consult to be sent, that patient is sitting at home now, he’s been post-op for five days, and maybe he needs care, so, what is timely initiation of care? – HHC Coordinator, Site 1.

Notably, all Veterans interviewed felt that their needs were met during their transition from hospital to home:

They got all, all kinds of programs of transportation, which they’ve helped me get since. They have, if you need food, they’ve got places we could go to and everybody would bring it to us and they pretty much cover a lot of it… – Veteran, Site 2.

Meanwhile, some VHA and HHC clinicians voiced issues contacting Veterans with social needs to follow up after discharge and organize HHC visits:

Some of our Veterans don’t have cellphones or where they live is in rural areas where they don’t get good cell services, so I know I get view alerted [in the medical record] sometimes where the community nursing or home health agency have had trouble contacting the Veterans to schedule a time to come out. – VHA Physician, Site 4.

### HHC delivery

Once HHC is initiated, coordination between VHA and HHC clinicians was lacking in knowledge of social needs resources and addressing order issues that delay Veteran care. Yet, HHC agencies have the valuable perspective of being able to identify social needs that may not be visible in the hospital:

I think those social determinants are actually helped by home health rather than hurt by home health… so that you have a more realistic picture of what the patients looks like in their own environment. – VHA Physician, Site 4.

Lack of closed-loop communication is a common barrier described by HHC agencies to address identified social needs. Multiple HHC agencies described instances where they detected social needs for a Veteran but were unable to follow up or easily alert the VHA of these needs:

We call and we don’t really know what happens, like it’s not like the social worker is reaching out to our people. The social worker may reach out to the patient and may do something with the patient, but… unless if we’re calling them back to say where are we at with this, sometimes we don’t know. – HHC Director, Site 4.

In addition to being unable to follow up on Veterans’ social needs, some HHC clinicians felt they had untapped resources that could benefit Veterans, but they were unable to use due to VHA policy and benefit coverage. One example from a HHC coordinator described the inability to use their social worker when they identify a Veteran with social needs since HHC social work services are not reimbursed by the VHA.

Both Veterans and care partners described times when they had social or medical needs after discharge with HHC, but experienced delays or unmet needs. One care partner called their HHC agency instead of the VHA when experiencing emergent medical issues due to delays in Home-Based Primary Care (i.e., VHA program that provides health care services to Veterans in their home who need team based in-home support for ongoing diseases) initiation and not having a VHA point of contact. They also described needing additional home support after their Veteran’s hospitalization, but services were not available for several weeks after discharge when they were no longer needed.

Another Veteran experienced housing and food insecurity after discharge and their HHC nurses went above and beyond to address their social needs. In this situation, the VHA was not contacted to leverage resources that could have supported this Veteran:

I was living under bridges before I met these people, and [HHC Nurses] helped me get established in a hotel room. They helped take care of my health. They made sure I had everything that I needed whether it was medical or food or clothing, ‘cause they told me where I could go to get clothing, and they told me where I could get food. They gave me numbers to churches that came and helped me. I mean, I speak very highly of them because they were all very nice. – Veteran, Site 4.

## Discussion

Our study findings suggest opportunities for VHA to collaborate with HHC to detect and address social needs for Veterans receiving care in the VHA and the community. VHA clinicians recognize the benefit of HHC in social needs assessment, but, at present, lack care coordination infrastructure and processes to leverage HHC workers’ knowledge about social needs.

Throughout the hospital to home with skilled HHC transition process, lack of communication between HHC and the VHA can contribute to challenges accessing care, delays in care, and unmet social needs. HHC agencies who meet with the Veterans at their home may identify Veterans’ social needs but lack a pathway to alert the VHA and tap into VHA resources. An additional challenge is that some community hospitals and HHC agencies may not have knowledge of VHA benefits which results in delayed Veteran care and issues with care coverage. This knowledge gap can affect Veterans’ ability to access VHA’s social needs resources.

Similar to our findings, challenges with care coordination and communication between VHA and community care exacerbate when Veterans have social needs such as housing instability, lack social support, and live in rural areas [22, 23]. Franzosa et al. reported similar themes from their study of home health aides that illustrated the complexities of establishing HHC, lack of formal communication channels between HHC agencies and VHA, and HHC workers as an important source of patient information [24]. Other researchers reported barriers to effective communication without standardized processes [11, 12]. Although there are established challenges for VHA and community care coordination, less is known about how this process affects the ability to meet Veterans’ social needs and how programs might be adapted to address these issues.

To effectively address Veterans’ social needs using HHC’s unique knowledge, the care coordination process between VHA and HHC needs to be standardized with a closed-loop communication pathway across the entire ecosystem. Until this pathway is developed, consistent reporting and addressing of social needs across care settings will remain a barrier. While the VHA is an innovation hub, there are community healthcare system interventions that could be adapted for the VHA ecosystem to address these barriers. Process innovations in community settings show promising results to improve care coordination and communication issues between healthcare systems and HHC. THRIVE, a clinical pathway developed to support individuals insured by Medicaid in the transition from community hospital to HHC, includes community health workers who address patients’ social needs and has shown preliminary reductions in 30-day readmission and emergency department use compared to baseline [8]. Industry partnerships continue to grow with SDOH information exchanges working with health plans, state governments, and healthcare systems, such as ChristianaCare’s partnership with Unite Us, to provide geographically tailored social needs resources for patients [25–27]. Such innovations hold promise for their ability to be adapted and piloted to create a robust VHA pathway to detect and address Veterans’ social needs during care transitions.

The VHA developed promising programs such as Care Coordination and Integrated Case Management and the Transitions Nurse Program which contribute to standardized care coordination but are not solely focused on community care, HHC, or social needs [15, 28]. The Office of Health Equity is piloting a social risk screening and referral tool through the Assessing Circumstances & Offering Resources for Needs (ACORN) Initiative across diverse clinical settings [29]. ACORN social risk domains include food, housing, utilities, transportation, education, employment, legal needs, interpersonal violence, and social isolation. While ACORN has mainly been piloted in outpatient settings, some inpatient settings have adopted the screening tool. Future research and QI programs should work to understand which social needs are most important to screen for at discharge, who is best to communicate that information, and a pathway to address these social risk factors [5]. VHA researchers and operational partners should evaluate existing care coordination and SDOH programs, both in the VHA and community, to understand their core components and how they can be integrated to develop a comprehensive social risk care transitions pathway.

Since opportunity comes with modernization, the VHA transition to a new electronic health record (EHR) system has potential to optimize communication and information exchange with HHC [30]. EHR innovation could provide a new platform to screen and address Veterans’ social needs. Informed by VHA and community programs, there are a few ways the VHA could partner with HHC to address social needs using technology and the EHR. Ideally, an application or platform could be developed that allows HHC clinicians to access the ACORN screening tool and communicate with VHA clinicians using EHR integration. To address the lack of education for community care clinicians, the Office of Integrated Veteran Care could offer trainings and resources on VHA benefits and resources to HHC agencies, community hospitals, and skilled nursing facilities. Another opportunity could be partnering with an SDOH information exchange to give VHA and HHC clinicians the tools to have tailored resources for Veterans easily accessible [26]. These relationships may increase VHA accessibility through collaborative process improvement to reduce delays in Veteran care. Insights from these interviews may inform future research and operational development to support Veterans’ hospital to HHC transition that supports social needs detection in the home. While this research is preliminary, we recommend that operational partners consider two steps forward: (1) develop closed-loop communication and education pathways between VHA and HHC and (2) develop a partnership to adapt and integrate the ACORN social risk screener into HHC pathways.

### Limitations

This study has several limitations. We conducted a secondary analysis of interviews for which the primary purpose was pre-implementation planning for a QI intervention that included questions about addressing social needs through the hospital to home with HHC transition. The parent study analyzed these interviews to identify barriers and facilitors to TNP-HHC implementation. The secondary analysis was conducted due to important social needs data detected in the interviews that was not the focus of the primary analysis [31]. While this is a secondary analysis of that data, all researchers and analysts on this project were TNP-HHC study team members and the data were analyzed within a similar timeframe to the parent study. Questions about Veteran social needs were included in the interview guide as a secondary interest of the parent study.

We had limited Veteran demographic data and were unable to report the demographics of care partners and clinicians as this information was not collected. Due to the parent study design, the goal was the representativeness of roles involved in post-acute care transitions instead of saturation. We had both passive and active declines but do not think these differences warrant significant concern about the results since Veteran populations can be hard to reach and recruitment was conducted during COVID. Clinicians have high workloads and may not have felt their role appropriate for the interview. However, we followed COREQ guidelines throughout the process to optimize the robustness of this analysis [14]. Finally, this study used a convenience sample at four VAMCs and these results may not be generalizable. These results may not include all perspectives of those involved in the hospital to home with HHC transition. Strengths of our study include its inclusion of Veterans, care partners, VHA and HHC clinicians. In addition, this study was conducted by a multidisciplinary team of clinicians, health services researchers, qualitative analysts, methodologists.

## Conclusion

HHC is an underexplored space for social needs detection in the VHA with the ability to access Veterans who receive care in the VHA and the community. Due to process barriers for communication and knowledge of the VHA, HHC clinicians face barriers to report detected social needs to the VHA and facilitate Veterans’ connection to necessary resources. While this research is preliminary, we recommend two steps forward from this work: (1) develop closed-loop communication and education pathways with HHC and (2) develop a partnership to integrate a social risk screener into HHC pathways.

### Electronic supplementary material

Below is the link to the electronic supplementary material.

Supplementary Material 1

## Data Availability

Data sharing is not applicable to this article as no datasets were generated or analyzed during the current study.
