# Supplementary material for: Perspectives on supporting Veterans’ social needs during hospital to home health transitions: findings from the Transitions Nurse Program
Source: BMC Health Serv Res. 2024 Apr 25;24:520. doi: 10.1186/s12913-024-10900-9 (PMC11043030; doi:10.1186/s12913-024-10900-9)
Supplement: Supplementary file 1 — Supplementary Material 1 [file 12913_2024_10900_MOESM1_ESM.docx]

**VA Clinician Interview Guide**

*Grounded prompts: If responses are limited or require clarification, probes may be used to elicit more detailed responses. Probes should use words or phrases presented by the participant using one of the following formats:*

*1. What do you mean by ____________.*

*2. Tell me more about ____________.*

*3. Give me an example of ____________.*

*4. Tell me about a time when ___________.*

**Goal of the interview:** *To understand: (1) Veteran, caregiver, and clinician experience with coordinating care following discharge from VAs with HHC and (2) Context, current process state, and perceived issues implementing TNP-HHC program.*

**Structure***: Semi-structured, use a case example to help Clinicians dive deeper into the topic and probe on context, process, perceived issues and solutions. If interviewee says “I don’t know” ask them to 1) refer us to someone who does 2) continue on with the interview.*

1. Tell me about your role in the VA.
2. How long have you been working at the VA?
3. Tell me about the process of coordinating home healthcare services for Veterans following acute hospitalization.
   1. Can you tell me about a specific time when you referred a Veteran to receive home healthcare services? (Recent or notable experience)

Probes

- - 1. What went well during this process?
    2. What challenges did you encounter?

1. In general, what type of information did you need to coordinate home healthcare services for your Veterans?
   1. What does your communication with other clinicians or administrators look like? (probe on VA and non-VA clinicians)
   2. What information do wish you knew in sending patients home following hospital discharge?
   3. What would be the easiest way to obtain this information?
2. How do you communicate with Veterans and caregivers, your decision to send Veterans home with a home health care service order?
   1. Can you think of a recent or memorable discussion with your Veteran patient/caregiver as an example?
      1. What went well during these discussions?
      2. What challenges did you encounter? What did you do about that?
3. How are home health care agencies informed or involved once the decision to send Veterans with home healthcare is made?
4. Tell me what a well-coordinated home health care looks like for your Veteran patients.
5. Tell me what a challenging home healthcare coordination looks like for your Veteran patients.
   1. What solutions do you suggest for addressing some of these challenges? Or if you had a magic wand, what would you do about these?
6. How has COVID-19 impacted the coordination around home health care services for Veterans?
7. What are some social determinants of health needs you observe during this home health care coordination?
   1. Do you think the VA is addressing some of these needs?
   2. How could we better address these needs?
8. We are interested in learning about your experience with home healthcare service coordination because we would like to improve the current process. We are in the process of implementing a care coordination program aimed at improving care coordination for Veterans discharged home with home healthcare services. You should have received a one-pager on this program prior to the interview.
   1. What are your general thoughts about such a program?
   2. What do you think will be our biggest challenge in implementing this program?
   3. How do you think such a program could work well?
   4. What would make it integrate well with your current processes?
   5. What would be important to you to know in order to evaluate the impact of the program?
   6. Who do you think we should involve in the planning and implementation process of such a program?
   7. Are there other high-risk patients you believe could benefit from such a program?
      1. ***What about patients with dementia and their caregivers? What kinds of support will they need?***
   8. What key outcomes should we measure (probe: readmissions, other utilization outcomes, etc.)
   9. What would make this program sustainable at your VAMC?
9. Is there anything else we have not asked you about that you would like to share?

Thank you for your participation in this study.

**Non-VA HHC Clinician Interview Guide**

*Grounded prompts: If responses are limited or require clarification, probes may be used to elicit more detailed responses. Probes should use words or phrases presented by the participant using one of the following formats:*

*1. What do you mean by ____________.*

*2. Tell me more about ____________.*

*3. Give me an example of ____________.*

*4. Tell me about a time when ___________.*

**Goal of the interview:** *To understand: (1) Veteran, caregiver, and clinician experience with coordinating home health care for Veterans following hospital discharge and (2) Context, current process state, and perceived issues implementing TNP-HHC program*

**Structure***: Semi-structured, use a case example to help Clinicians dive deeper into the topic and probe on context, process, perceived issues and solutions. If interviewee says “I don’t know” ask them to 1) refer us to someone who does 2) continue on with the interview.*

1. Tell me about your role at [name of non-VA clinic].
2. How long have you been working at the [name of non-VA site]?
3. How long have you been in your current role?
4. Tell me about your involvement with providing home health care services to Veterans
5. What does the process of providing Veterans home health care services look like?
   1. Can you tell me about a specific/the last time when you coordinated home healthcare service to a Veteran? (Recent or notable experience)

Probes

- - 1. What went well during this process?
    2. What challenges did you encounter?

1. In general, what type of information did you need to coordinate home healthcare services to your Veteran patients?
   1. What does your communication with VA clinicians or administrators look like?
   2. What information do wish you knew in coordinating home healthcare services to Veterans?
   3. What is the best way to get that information to you?
2. How are you informed by the VA once the decision is made to send Veterans home with home healthcare services?
   1. In your opinion, what are the VA facilities doing well?
   2. In your opinion, what do you think the VA should work to improve on?
3. How do you communicate your decision to provide home healthcare services with Veterans and their caregivers?
   1. Can you think of a recent or memorable discussion with your Veteran patient/caregiver as an example?
      1. What went well during these discussions?
      2. What challenges did you encounter?
4. Tell me what a well-coordinated home health care transition looks like for your Veteran patients.
5. Tell me what a challenging home healthcare coordination looks like for your Veteran patients.
   1. What solutions to you suggest for addressing some of these challenges?
6. Tell me about some of the social determinants of health needs you see of our Veteran patients
   1. How do you address those needs when you see them?
   2. Is there anything you believe the VA can do to address these needs?
7. How has COVID-19 impacted the coordination around home healthcare services for Veterans?
8. We are interested in learning about your experience with home healthcare service coordination because we would like to improve current process. *We are implementing a care coordination Program aimed at improving care coordination for Veterans discharged home with home healthcare services. We sent you a one pager after we scheduled the interview. (see if they received it)*
   1. What are your general thoughts about such a program?
   2. What do you think would be our biggest challenge?
   3. What could we do to overcome this challenge?
   4. How do you think such a program could work well?
   5. How could it integrate best with your current processes?
      1. What would be important to know to understand the impact of the program?
   6. Who do you think we should involve in the planning and implementation of such a program?
   7. Are there specific high-risk patients you believe could benefit from such a program?
      1. ***What about patients with dementia and their caregivers? What kinds of support will they need?***
   8. What key outcomes should we measure (probe: readmissions, other utilization outcomes, etc.)
   9. What would make working with VAMCs sustainable? Or How could we make it last long-term?
9. Is there anything else we have not asked you about that you would like to share?

Thank you for your participation in this study.

**HHC Veteran Interview Guide**

*Grounded prompts: If responses are limited or require clarification, probes may be used to elicit more detailed responses. Probes should use words or phrases presented by the participant using one of the following formats:*

*1. What do you mean by ____________.*

*2. Tell me more about ____________.*

*3. Give me an example of ____________.*

*4. Tell me about a time when ___________.*

**Goal of the interview:** Understand patients’ reflections on the experience of transitioning with a home healthcare service. Focus on the transitions & coordination surrounding home healthcare services.

**Structure**: Semi-structured, only probe if the patient doesn’t naturally bring up the topic in conversation. Use the guide as a guide; jump around to various questions in the guide as interview topics naturally occur and re-phrase questions as needed in order to maintain a conversational tone.

1. How long you have been receiving home healthcare services?
2. How is home healthcare service supporting you?
3. How was the process of arranging home healthcare service prior to leaving the hospital?
4. How did you choose your particular home healthcare provider?

**Probes**

- 1. Did you feel you had a choice?
  2. Was any information presented to you on options for home health care?
  3. What was important for you when deciding on a home healthcare agency?
  4. What has been surprising or different than you expected?
     1. If nothing; how has it met your expectations?

1. What were the goals you were hoping to accomplish when you left the hospital?

**Probes**

- 1. How have those goals changed since being home with home healthcare? (probe on each goal)

1. Knowing what you know now, would you have made the decision to get home healthcare services?

**Probes**

- 1. Why? Why not?
  2. What information do you wish you had/knew about home healthcare services?

1. Did the VA work with the home healthcare agency to make sure you were followed up once you got home?
   1. Is there anything that went particularly well?
   2. Anything that went particularly poorly?
   3. Is there anything you wish you knew beforehand about the VA process?

SDOH focused questions: we are interested in also learning about conditions that might impact your health such as physical, social, environmental and financial factors you have been experiencing following hospital discharge. The following questions focus on that area.

1. Tell me about some unmet needs you might have experienced following hospital discharge. Probe: availability of resources to meet daily needs, living wages, healthy foods, experience of discrimination, social support and social interactions, transportation.
   1. If some challenges are described, probe on how often they experienced these unmet needs [use participant language to probe]
2. Where do you go to get these resources when you experience these unmet needs?
   1. How helpful have the VA been?
   2. What do you wish they have done to address your needs?
3. We are in the process of implementing the **Transitions Nurse Program** aimed at improving care coordination for Veterans discharged home with home healthcare services. This is a care coordination program that will help Veterans receive the care they need after they leave the hospital.
   1. What are your general thoughts about such a program?
   2. What do you think will be our biggest challenge in making sure this program works for our Veterans?
   3. Do you have any suggestions as to making a program like this work well?
   4. Who do you think we should involve in making sure this program will work well?
4. Is there anything else you would like to share with me about your experience being discharged home with home healthcare services?
5. Besides your medical team, could you please tell us who is the one person who helps you most with your health? (to identify Veterans caregivers)

Thank you for your participation in this study.

**HHC Caregivers Interview Guide**

**Goal of the interview:** *Understand caregivers’ reflections on the experience of home healthcare services for their loved ones following hospital discharge.*

**Structure***: Semi-structured, only probe if the caregiver doesn’t naturally bring up the topic in conversation. Use the guide as a guide; jump around to various questions in the guide as interview topics naturally occur and re-phrase questions as needed in order to maintain a conversational tone.*

1. Can you tell me about your relationship to your _[name of the patient] (*mention relationship e.g.: mother*) __?

***Probes***

- 1. Can you tell me about the kind of support or care you provide?
     1. Has this changed since [patient’s name] has been home from the hospital? If so, how?

1. Can you describe why your _ (*mention relationship e.g.: mother*) __was sent home with home healthcare services recently?

***Probe***

- 1. What was the goal of the home healthcare service?

1. What do you think of the care being provided by the home health care service?
   - 1. Is the HHC services the same/different from what was initially described?
     2. Tell me about the best part of having HHC service
     3. Tell me about the worst aspects? (Probe: scheduling, convenience, staffing, etc.)
2. What was the experience following discharge for your _ (*mention relationship e.g.: mother*) ___ from the hospital to home with home healthcare service?

***Probes***

- 1. Is this the first time your _ (*mention relationship e.g.: mother*) ____ has been with a home healthcare service?
     1. *If not first time*: How did your previous experience with home healthcare services help shape your decision/experience?
  2. How was the decision made to send your _ (*mention relationship e.g.: mother*) ____ home with this particular home healthcare agency made?
     1. What things did you consider when making this decision?
     2. Who talked with you about the HHC option?
     3. Did you agree with this decision?
     4. Were there other options you were both looking at for care following hospitalization?
     5. How were the other options presented to you?
     6. Did you think that presentation of information went well?

1. Knowing what you know now, would you have made this decision differently?
2. Tell me about some unmet needs (social, physical or environmental) your (*mention relationship e.g.: mother*) might have experienced following hospital discharge. Probe: availability of resources to meet daily needs, living wages, healthy foods, experience of discrimination, social support and social interactions, transportation.
   1. If some challenges are described, probe on how often they experienced these unmet needs [use participant language to probe]
3. Where do you go to get these resources when you experience these unmet needs?
   1. How helpful have the VA been?
   2. What do you wish the VA has done to address these unmet needs?
4. ***We are in the process of implementing*** ***the Transitions Nurse Program aimed at improving care coordination for Veterans discharged home with home healthcare services. This is a care coordination program that will help Veterans receive the care they need after they leave the hospital.***
   1. What are your general thoughts about such a program?
   2. What do you think will be our biggest challenge in making sure this program works for our Veterans?
   3. Do you have any suggestions for making a program like this work well?
   4. What do you think could go wrong with a program like this?
   5. Who do you think we should involve in making sure this program will work well?
5. Is there anything else you would like to share with me about your _ (*mention relationship e.g.: mother*) _ experience being sent home with home healthcare after being hospitalized?

Thank you for your participation in this study.
